# Convolutional Neural Network-Based Age Prediction from Cephalometric Images and Analysis of Site-Specific Associations

**DOI:** 10.3390/jcm15145404

**Published:** 2026-07-09

**Authors:** Toshiro Emori, Ryo Hamanaka, Runa Yamaguchi-Higuchi, Yui Horiguchi-Nakayama, Sayaka Iwata, Kazuhiro Ogawa, Arina Kitaura, Hiroya Komaki, Jun-ya Tominaga, Noriaki Yoshida

**Affiliations:** 1 Department of Orthodontics, Nagasaki University Hospital, 1-7-1 Sakamoto, Nagasaki 852-8588, Japan; bb55322205@ms.nagasaki-u.ac.jp (T.E.); yamaguchi.ortho@outlook.jp (R.Y.-H.); 2 Department of Orthodontics and Dentofacial Orthopedics, Nagasaki University Graduate School of Biomedical Sciences, 1-7-1 Sakamoto, Nagasaki 852-8588, Japan; info.hamanaka@gmail.com (R.H.); bb55322213@ms.nagasaki-u.ac.jp (Y.H.-N.); bb55323202@ms.nagasaki-u.ac.jp (S.I.); bb55324202@ms.nagasaki-u.ac.jp (K.O.); bb55324206@ms.nagasaki-u.ac.jp (A.K.); tomi226@nagasaki-u.ac.jp (J.-y.T.); nori@nagasaki-u.ac.jp (N.Y.)

**Keywords:** craniofacial growth prediction, lateral cephalometric radiographs, convolutional neural network, ResNet50, ConvNeXt-T

## Abstract

**Background/Objectives:** Accurate prediction of craniofacial growth is essential for establishing orthodontic diagnoses and planning treatment. However, the ultimate extent of jaw growth remains largely judged subjectively based on clinical experience. In this study, we developed a convolutional neural network (CNN) model to predict chronological age from lateral cephalograms and to investigate whether artificial intelligence (AI) can autonomously learn growth-related morphological features. We also reevaluated which anatomical regions are most informative for predicting growth. **Methods:** We retrospectively analyzed 2116 cephalograms from patients 5–30 years old with malocclusion. After excluding patients with craniofacial syndromes or systemic diseases, 2014 images were used for training and 102 for testing. The mean age of the training dataset was 19.51 years (standard deviation [SD]: 4.71), whereas that of the test dataset was 18.20 years (SD: 7.33). In addition to the entire cephalograms, five regional datasets were generated (mandible, maxilla, cervical vertebrae, frontal region, and cranial base). All images were resized to 256 × 256 pixels and trained with a ResNet50-based CNN. Performance was evaluated using mean absolute error (MAE), Pearson’s correlation coefficient (*r*), and coefficient of determination (*R*^2^). To assess growth-related learning, test data were divided into growth (5–19 years) and post-growth (20–30 years) groups, and age trends were analyzed using a sliding window approach with an 8-year window. **Results:** The model trained on entire cephalograms achieved high accuracy in the younger group (MAE 1.16 years, *r* 0.952, *R*^2^ 0.884), but performance declined markedly in the older group. Among the regional models, accuracy was highest for the mandible, followed by the cervical vertebrae and maxilla. **Conclusions:** The CNN model predicted chronological age with high accuracy, particularly in patients <20 years old, and may have captured age-associated craniofacial features related to growth. Prediction was most accurate in the mandible and cervical vertebrae—regions clinically used to assess growth. As a proof of concept, this approach may provide a foundation for future studies of craniofacial growth prediction through transfer learning, although further validation is required.

## 1. Introduction

Accurate prediction of the magnitude, direction and timing of completion of craniofacial growth is essential for establishing appropriate diagnoses and planning treatment in growing patients. Treatment strategies can vary substantially depending on the estimated direction of growth. For example, a dolichofacial growth pattern is characterized by predominantly downward growth of the mandible. In these patients, molar distalization should be avoided to prevent additional clockwise rotation of the mandible due to the wedge effect. The likelihood of non-extraction treatment might thus be decreased in such cases. In contrast, a brachyfacial pattern is characterized by predominantly forward growth of the mandible with minimal rotational change, so the possibility of non-extraction therapy might be increased in these patients.

In addition, the timing of treatment initiation in patients with skeletal disharmony depends on how long growth continues. If skeletal mandibular protrusion is anticipated due to late-stage growth, early intervention should be avoided where possible. Otherwise, surgical orthodontic treatment may be required due to relapse after early orthodontic treatment. In contrast, when growth predictions suggest that growth potential is minimal, unnecessary orthognathic surgery can be avoided with early intervention.

Various radiographic methods have been employed to assess skeletal maturity for the purpose of predicting craniofacial growth. However, these approaches are associated with inherent limitations in both the accuracy and reproducibility of growth prediction [1,2,3,4,5,6,7]. Among them, the hand–wrist maturation (HWM) and cervical vertebral maturation (CVM) methods are two of the most widely utilized techniques for evaluating skeletal maturity. The HWM method is useful for assessing the current stage of skeletal maturation, but its ability to precisely predict the timing of the pubertal growth spurt is limited due to interindividual variability and the cross-sectional nature of the assessment. Consequently, the onset of the pubertal growth spurt cannot be reliably predicted [1,8]. A notable drawback of the HWM method is that additional radiation exposure is required in addition to lateral cephalometric radiographs.

In contrast, the CVM method allows cephalometric images taken for orthodontic diagnosis to be repurposed for assessing skeletal maturity. This advantage has contributed to the widespread application of this method in orthodontic practice [4,5,9,10,11]. Nevertheless, the CVM method shows potential methodological errors due to inter- and intra-observer variability. Gabriel et al. [12] reported that even after training 10 private orthodontists in the CVM method, inter-examiner agreement for CVM staging was less than 50%. They concluded that accurate CVM evaluation requires an advanced level of understanding and expertise in cervical vertebral anatomy. Further, numerous studies have examined the reliability of the CVM method through comparisons with other indicators of skeletal maturity, including HWM [10,13]. Consequently, whether the cervical vertebrae constitute the most appropriate anatomical site in the craniofacial region for assessing skeletal maturity remains contentious.

Such inter- and intra-observer variability may potentially be mitigated through the application of convolutional neural networks (CNNs), which have rapidly advanced in recent years. CNNs are capable of automatically learning and extracting hierarchical features from images and have driven substantial progress in the application of artificial intelligence (AI) technologies within the medical field [14,15,16,17]. In the shallow layers, CNNs typically learn simple patterns such as edges and line segments, whereas deeper layers capture more complex structures, including detailed maxillofacial morphology and higher-order features related to skeletal maturation. CNNs have already been introduced to the CVM method for the assessment of bone maturity [18,19,20]. Because most craniofacial structures visible on cephalograms undergo morphological changes during growth, these alterations suggest that anatomical regions beyond the cervical vertebrae may also contain valuable information related to skeletal maturation. Despite this potential, studies applying CNNs to predict craniofacial growth using anatomical regions other than the cervical vertebrae remain limited. Furthermore, only a few studies have investigated whether CNNs can extract growth-related morphological features from the entire cephalometric image without relying on specific anatomical landmarks or regions, and further validation is therefore required.

In the present study, rather than focusing on specific anatomical regions such as the cervical vertebrae, the entire cephalometric image was utilized for model training. Further, by analyzing the craniofacial regions contributing to age prediction across different age groups, we aimed to identify region-specific patterns in the growth-related features learned by the model.

## 2. Materials and Methods

A retrospective observational study was conducted using lateral cephalometric radiographs obtained for the diagnosis of malocclusion at the Department of Orthodontics in Nagasaki University Hospital between 1 January 2008 and 31 July 2017. The study protocol was approved by the Nagasaki University Hospital Clinical Research Ethics Committee (approval no. 17082150; date of approval: 21 August 2017), and the requirement for written informed consent was waived because of the retrospective design. Instead, an opt-out approach was adopted, and information regarding the study was disclosed on the website of the Clinical Research Center to provide patients with the opportunity to refuse the use of their data. The study was conducted in accordance with the principles of the Declaration of Helsinki.

### 2.1. Patients and Dataset

**Figure 1:**
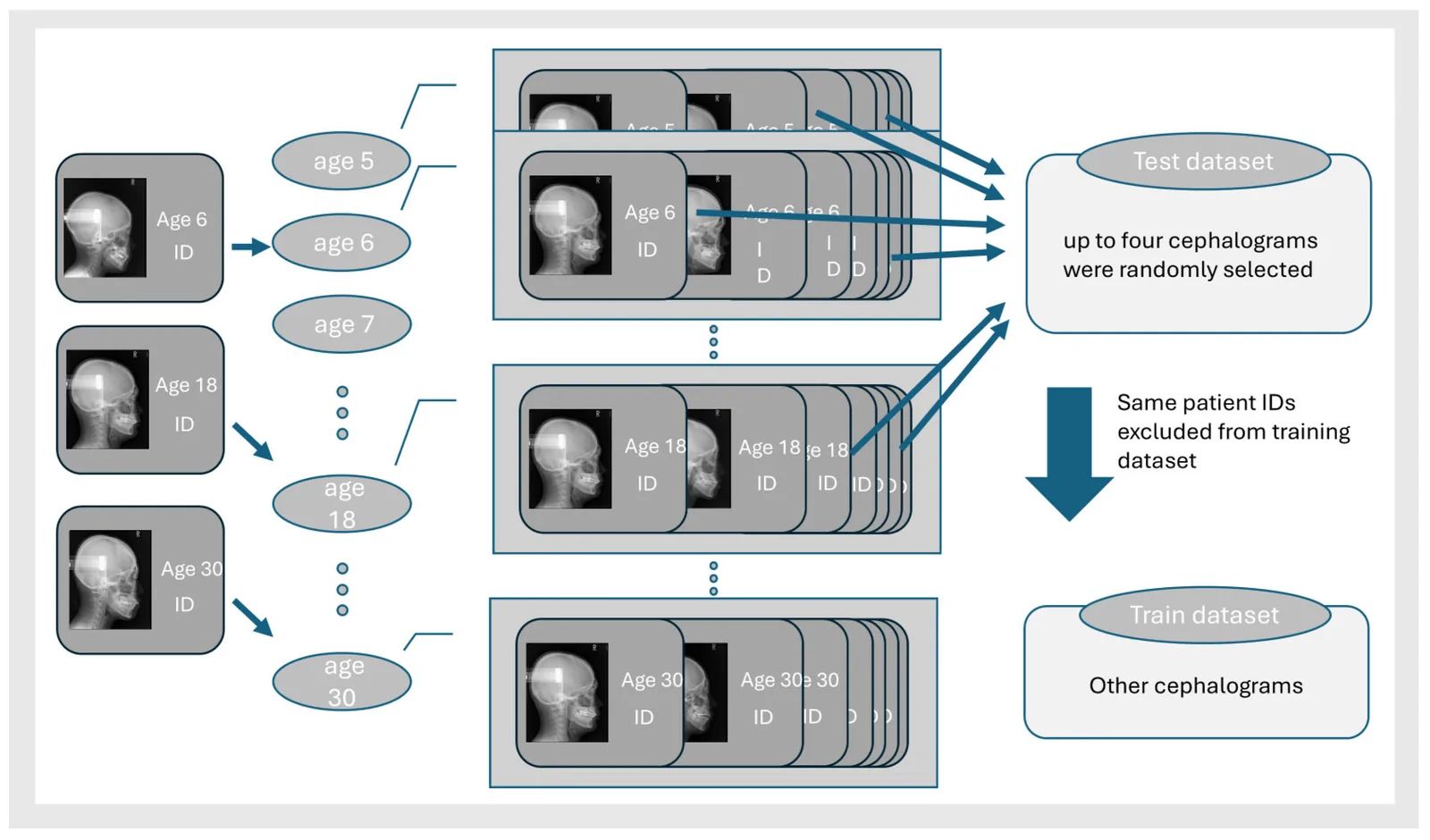
Construction of the training and test datasets. Cephalograms were first grouped according to chronological age. Up to four cephalograms were randomly selected from each age group for inclusion in the test dataset to achieve a balanced age distribution. To prevent data leakage, all cephalograms from patients included in the test dataset were excluded from the training dataset.

A total of 2116 cephalograms from 891 patients were collected from patients between 5 and 30 years old who visited Nagasaki University Hospital for orthodontic treatment between 1 January 2008 and 31 July 2017. Patients with syndromes or systemic diseases associated with craniofacial anomalies were excluded from the study. In addition, patients with skeletal Class III malocclusion requiring orthognathic surgery or severe craniofacial deformities were also excluded. The mean number of radiographs per patient was 2.37, and the median was 2. The test dataset was constructed to ensure a balanced age distribution, whereas sex balancing was not additionally applied. For each age, up to four cephalograms were randomly selected for the test dataset (Figure 1). The training dataset consisted of 827 individuals (303 males and 524 females), whereas the test dataset consisted of 64 individuals (21 males and 43 females). The mean age of the training dataset was 19.51 years (standard deviation: 4.71), whereas the mean age of the test dataset was 18.20 years (standard deviation: 7.33). Patients for whom multiple images were available were assigned to either the training or test dataset, but not both, based on patient ID. Accordingly, datasets were constructed such that each patient appeared in only one of the sets. A total of 2014 images were used for training and 102 images were used for testing.

To evaluate whether the model captured growth-related features, test data were stratified into two chronological age-based groups representing the growth and post-growth phases: 5–19 years (growth period) and 20–30 years (post-growth period). It should be noted that these classifications were based on chronological age rather than direct skeletal maturity assessment or cephalometric analysis.

### 2.2. Image Preprocessing and Model Development

#### 2.2.1. Resizing and Cropping Cephalograms

**Figure 2:**
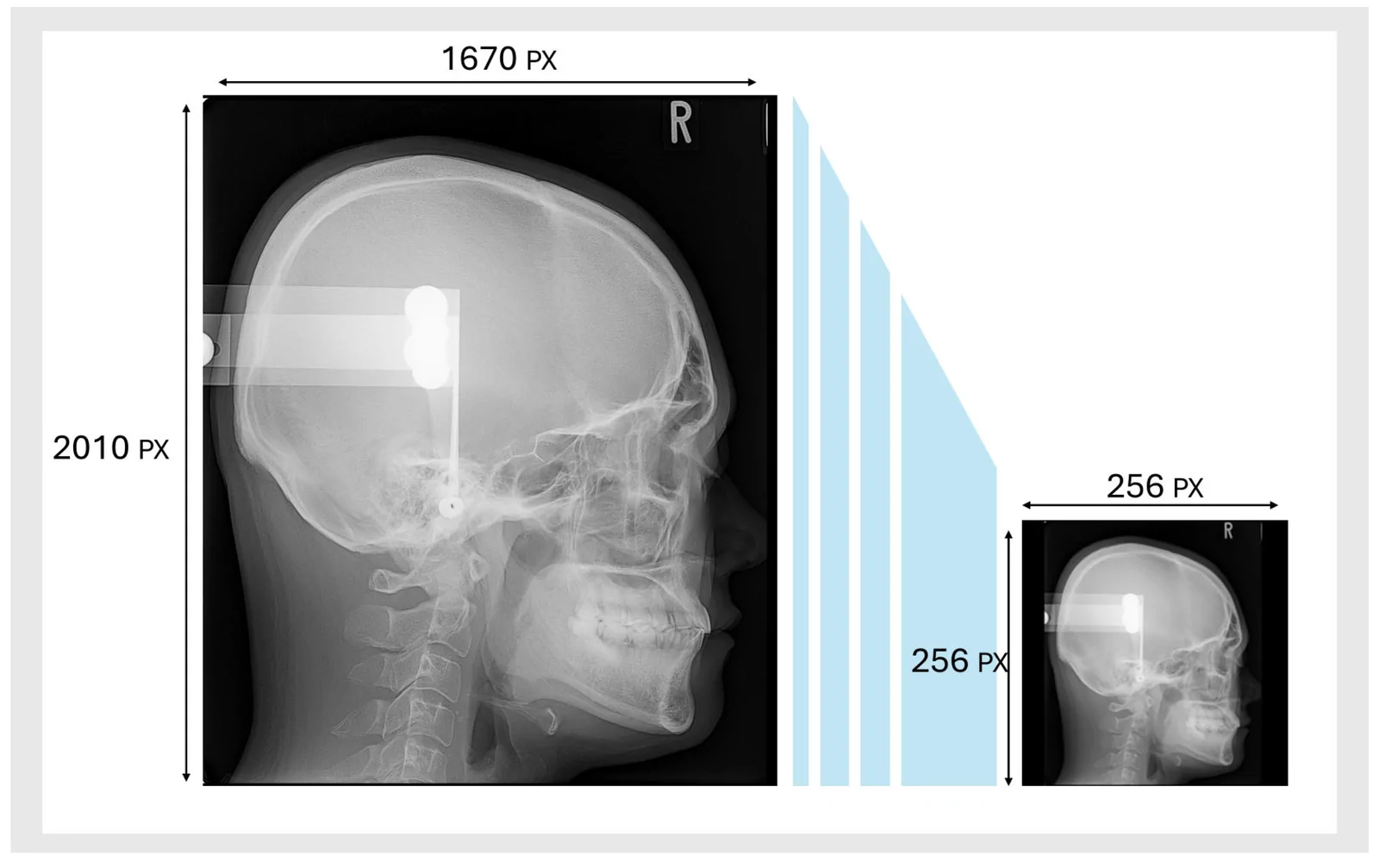
Preprocessing of cephalograms. Original cephalograms (1670 × 2010 pixels) were resized to 256 × 256 pixels by adding padding while preserving the aspect ratio for subsequent data augmentation.

All images were preprocessed to ensure compatibility with the two CNN architectures used for chronological age prediction, ResNet50 and ConvNeXt-T. Original cephalograms (1670 × 2010 pixels) were grayscale DICOM images and were converted to three-channel RGB images to match the network input requirements. The images were then resized to 256 × 256 pixels by padding while preserving the aspect ratio (Figure 2). Pixel intensities were converted to tensor format and normalized using fixed mean and standard deviation values. Because all cephalograms were acquired using the same radiographic device under standardized cephalometric imaging protocols and consistent imaging conditions, histogram equalization and contrast enhancement were not applied.

**Figure 3:**
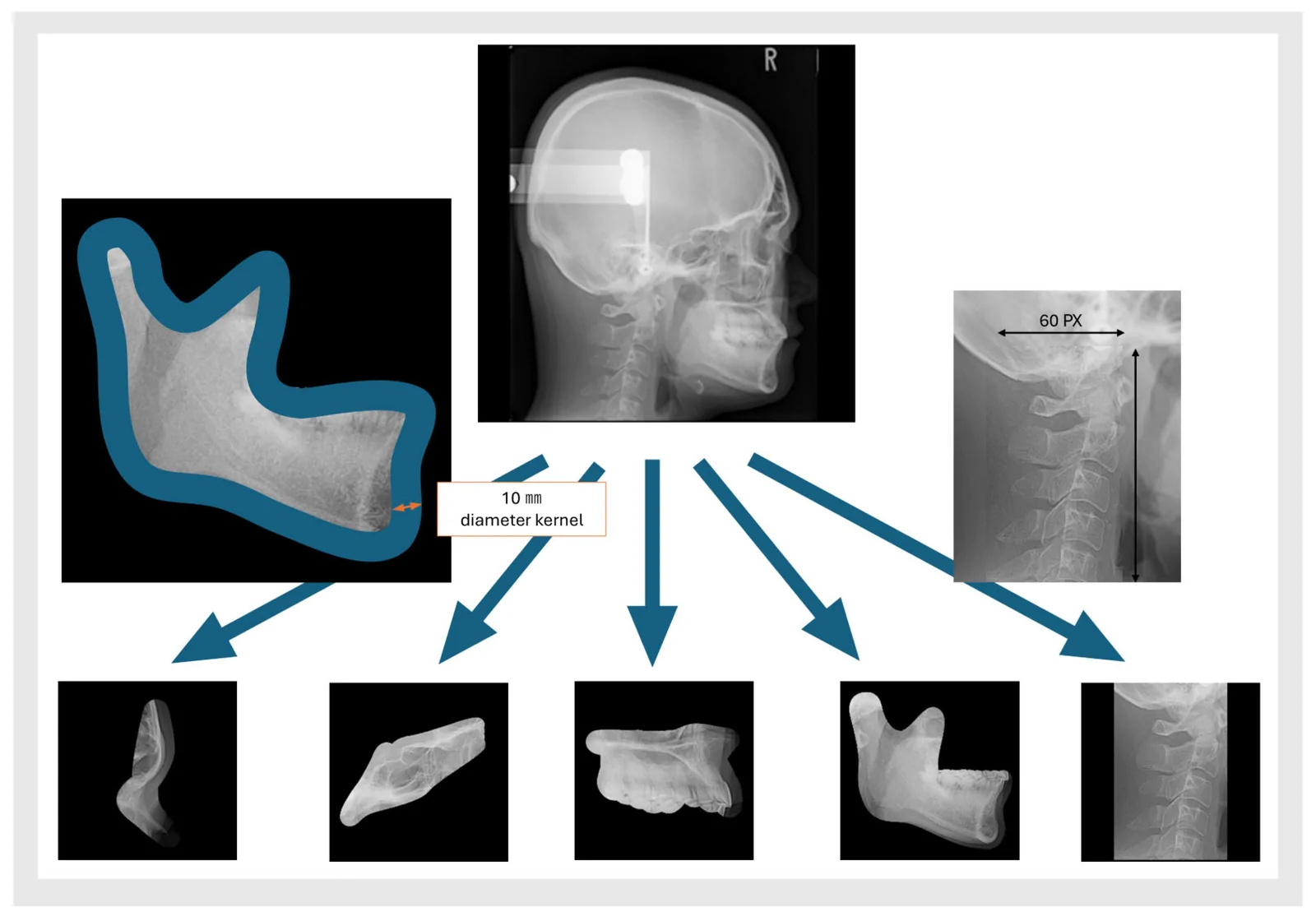
Region-based preprocessing of cephalograms. Five anatomical regions (mandible; maxilla; cranial base; frontal region; and cervical vertebrae) were cropped from each original cephalogram with a fixed margin, then resized to 256 × 256 pixels with padding.

To reduce overfitting, data augmentation was performed online during training using random affine transformations. Rotation, translation, and scaling were applied simultaneously to the training images. For ResNet50, random rotations (−2° to +2°), scaling (0.99–1.01), and horizontal/vertical translations (−2 to +2 pixels) were applied. For ConvNeXt-T, random rotations (−5° to +5°), scaling (0.99–1.01), and translations (−5 to +5 pixels) were used. To evaluate the contribution of specific craniofacial regions, five areas were defined: the frontal region (including the frontal sinus and glabella), maxilla, mandible, cranial base, and cervical vertebrae. For the frontal region, maxilla, mandible, and cranial base, a single operator manually masked each target structure using a brush of 10.0 mm in diameter, cropped the image 5 mm outside the masked boundary, and then padded and resized it to 256 × 256 pixels (Figure 3). The relatively large brush diameter was used so that minor variation in manual masking would have little effect on the results. The cervical vertebrae, which do not overlap with other structures, were extracted using a rectangular mask.

#### 2.2.2. Labeling

Chronological age was calculated from the date of birth of each patient and date of radiography and was used as the label. Prior to preprocessing and model training, the cephalograms were converted to PNG format, and personal information was removed from the images except for patient ID, date of birth, and imaging date, which were retained for dataset construction and age calculation. All images and the corresponding labels were stored in JSON format for training and testing.

#### 2.2.3. Model Training and Testing

All experiments were performed on a personal computer with an Intel Core i5-10400 CPU (Intel Corporation, Santa Clara, CA, USA) and NVIDIA GeForce RTX 2060 GPU (NVIDIA Corporation, Santa Clara, CA, USA). The CNN model was implemented using Python 3.8.10 and the PyTorch 2.4.1. For ResNet50, we used ReLU activation, the Adam optimizer, and a mean squared error (MSE) loss. The model was trained with a learning rate of 0.001. Weight decay was not applied. In the present study, ConvNeXt-T, a lightweight variant of ConvNeXt, was selected to match the number of parameters with ResNet50 and enable fair comparison. For ConvNeXt-T, we used GELU activation, the AdamW optimizer, and the MSE loss, with a learning rate of 0.0001, and a weight decay value of 0.05. The remaining training conditions were identical to those used for ResNet50. Both models were trained for 200 epochs with a batch size of 8. Pretrained ImageNet weights were not used. Random seed was fixed to 1234. A separate validation dataset and early stopping were not used. Epoch 200 was selected after confirming convergence of the loss curve. After training on 2014 images, both models were evaluated using the independent set of 102 test images. Because this study was designed primarily to investigate feature learning from cephalograms rather than to optimize predictive performance, a separate validation dataset, early stopping, and large-scale hyperparameter optimization were not used.

### 2.3. Performance Metrics and Statistical Analyses

Model performance was assessed using three statistical metrics: mean absolute error (MAE), representing prediction error; Pearson’s correlation coefficient (*r*), showing the correlation between predicted and actual values; and the coefficient of determination (*R*^2^), representing accuracy and reliability of prediction.

To investigate whether the model learned growth-related features, predictive performances were compared between the 5- to 19-year-old and 20- to 30-year-old age groups (Table 1). In addition, to explore performance trends across different age ranges, a sliding window analysis with an 8-year window was employed (Table 2). MAE was calculated as:

$$\mathrm{MAE}\;=\;\frac{\mathrm{sum of prediction error}}{\mathrm{number of test samples}}=\frac{\sum_{i=1}^{n}\left|y_{i}-y_{i}^{’}\right|}{n},$$

where $y_{i}$ is the actual age and $y_{i}^{’}$ is the predicted age. MAE represents the average magnitude of deviation between predicted and actual ages. A lower MAE indicates that predictions from the model are closer to the true chronological age, reflecting higher predictive accuracy.

Pearson’s *r* and *R*^2^ were calculated as:

$$r\;=\frac{\sum \left(y_{i}-\bar{y}\right)\left(y_{i}^{’}-\bar{y^{’}}\right)}{\sqrt{\sum {\left(y_{i}-\bar{y}\right)}^{2}}\sqrt{\sum {\left(y_{i}^{’}-\bar{y_{i}^{’}}\right)}^{2}}}$$

$$R^{2}=1-\frac{\mathrm{the sum of squared prediction errors}}{\mathrm{the sum of squared deviations of actual values from the mean}}$$

$$R^{2}=1-\frac{\sum_{i=1}^{n}{\left(y_{i}-y_{i}^{’}\right)}^{2}}{\sum_{i=1}^{n}{\left(y_{i}-\bar{y}\right)}^{2}},$$

where *r* quantifies the degree to which the predicted ages follow the trend of actual aging, with values near +1.0 indicating a strong correlation. Statistical significance was assessed using Student’s *t*-test under the null hypothesis that no correlation exists between predicted and actual ages. A *p*-value below the threshold indicates a significant correlation.

*R*^2^ represents the proportion of variance in the actual age explained by the model. Higher values (near +1) indicate stronger explanatory power. Negative values (R^2^ < 0) suggest that the model performed worse than simply predicting the mean age.

To quantify the uncertainty of the performance metrics, 95% confidence intervals (CIs) for MAE, Pearson’s correlation coefficient (*r*), and coefficient of determination (*R*^2^) were estimated using bootstrap resampling with 1000 iterations. Furthermore, differences in MAE between the ResNet50 and ConvNeXt-T entire models were evaluated using paired bootstrap resampling, and 95% CIs for the differences (ResNet50 − ConvNeXt-T) were calculated.

## 3. Results

### 3.1. Performance of the ResNet50 Model Trained on the Entire Cephalogram

**Figure 4:**
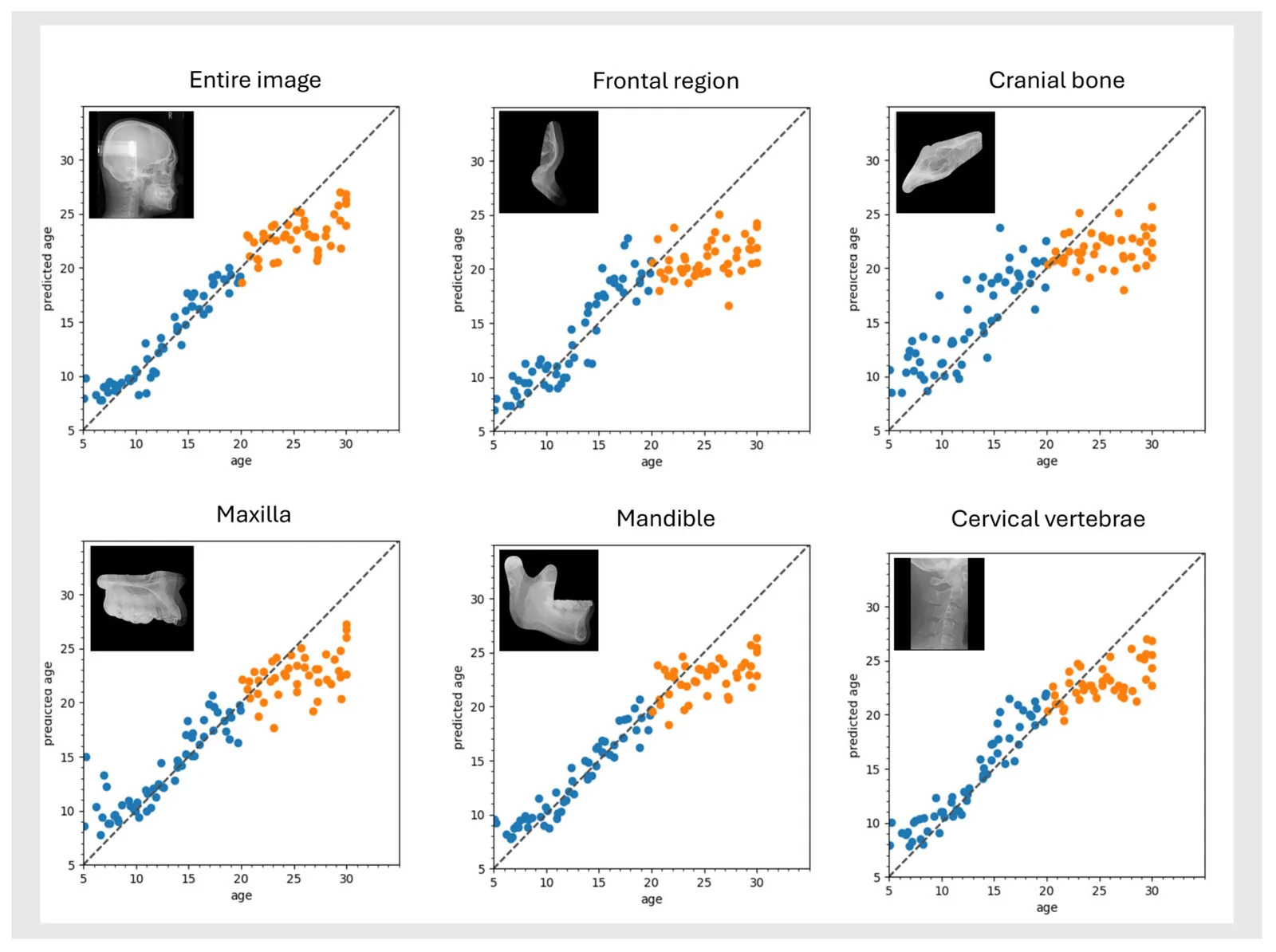
Distribution of age prediction accuracies for the entire region and specific anatomical region models. The x-axis represents the actual chronological age and the y-axis represents the predicted age. Blue dots indicate subjects <20 years old, and orange dots indicate subjects ≥20 years old. Points closer to the diagonal line (y = x) indicate higher predictive accuracy.

The predictive performance of the ResNet50 model when trained on the entire cephalogram was evaluated and compared between the <20- and ≥20-year-old test groups. *R*^2^ was 0.884 for the <20-year-old group and −0.221 for the ≥20-year-old group, indicating significantly higher predictive accuracy in the younger group (Figure 4). MAE was 1.17 years for the <20-year-old group and 2.73 years for the ≥20-year-old group, demonstrating lower prediction error in the younger group. Similarly, *r* was 0.952 for the <20-year-old group and 0.585 for the ≥20-year-old group, representing a markedly stronger correlation between predicted and actual values in the younger group. Collectively, these results indicate that the accuracy of chronological age prediction was consistently higher in the <20-year-old test group compared with the ≥20-year-old test group across all evaluated statistical metrics.

### 3.2. Performance of the ResNet50 Model Trained on Specific Anatomical Regions of the Cephalogram

Predictive performance of the ResNet50 model was compared across models trained on different regions of the cephalogram. In the <20-year-old group, *R*^2^ values in descending order were as follows: mandible, *R*^2^ = 0.883; cervical vertebrae, *R*^2^ = 0.774; frontal region, *R*^2^ = 0.741; maxilla, *R*^2^ = 0.709; and cranial base, *R*^2^ = 0.362 (Table 3). Notably, the model trained on the entire cephalometric image demonstrated the highest performance (*R*^2^ = 0.884), exceeding that of all region-specific models. In contrast, in the ≥20-year-old group, all region-specific models yielded negative *R*^2^ values, indicating worse accuracy of chronological age prediction (Table 3). These findings indicate that although region-specific models, particularly the mandibular model, show relatively high predictive performance during the growth period, use of the entire cephalometric image provides the most comprehensive and accurate representation of growth-related information.

In terms of MAE, the model trained on the mandibular region demonstrated the highest predictive accuracy in the <20-year-old group, with an error of 1.17 years, followed by the maxilla (1.52 years). The cervical vertebrae and frontal region showed MAE values similar to those of the maxilla. The poorest performance was observed for the cranial base, with an MAE of 2.77 years (Table 3). On the other hand, in the ≥20-year-old group, MAE for the model trained on the cervical vertebrae model showed the best performance, with an error of 2.78 years, followed by the mandible model with an error of 3.15 years. Regarding correlations, the frontal region showed a significant correlation between chronological and predicted ages (*p* = 0.011), whereas the cranial base did not show a significant correlation (*p* = 0.122).

The *r* value exceeded 0.8 for all regions in the <20-year-old group, indicating strong correlations, with *p*-values for all regions less than 10^−15^, showing significant associations between true and predicted values (Table 3).

Overall, region-specific models demonstrated lower predictive accuracy than the model trained on the entire cephalogram. In the <20-year-old group, the mandibular model achieved the highest performance among regional models, followed by the maxilla and cervical vertebrae. In contrast, all models in the ≥20-year-old group exhibited poor performance. Consistently, both the entire and region-specific models showed superior performance in the <20-year-old group compared with the ≥20-year-old group across all evaluation metrics, including *R*^2^, MAE and *r* (Figure 4, Table 3).

### 3.3. Comparative Analysis of Each Model by Age Group

**Figure 5:**
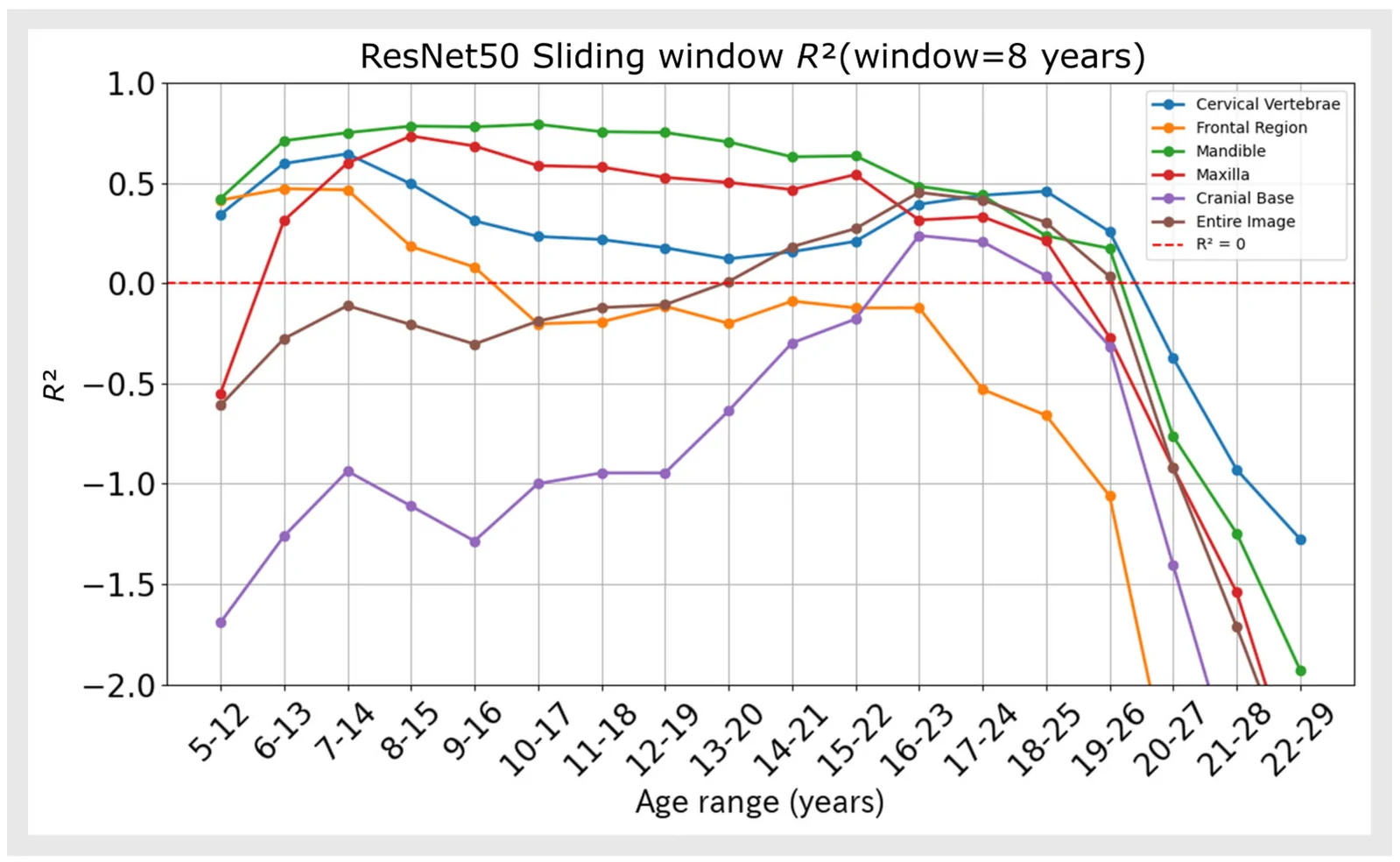
Sliding-window analysis of *R*^2^ for ResNet50 models trained on the entire and 5 specific regions of cephalograms. To examine model performance across different age ranges, *R*^2^ values were calculated using sliding 8-year windows. The x-axis represents age intervals, and the y-axis represents *R*^2^. Lines correspond to *R*^2^ values for the models trained on the entire and 5 specific regions, as indicated in the legend.

**Figure 6:**
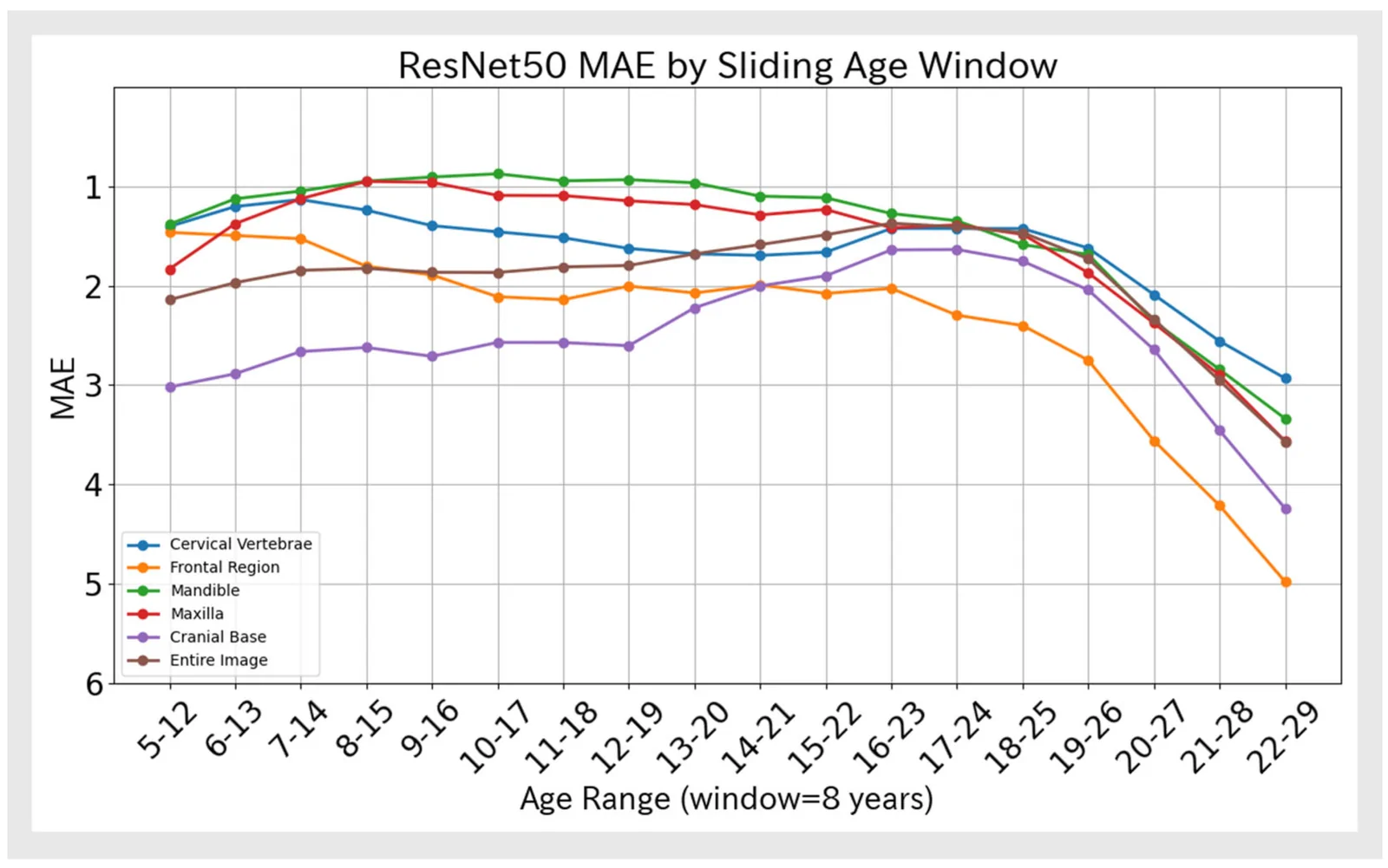
Sliding-window analysis of MAE for ResNet50 models trained on the entire and 5 specific regions of cephalograms across 8-year age intervals. The x-axis shows age ranges (8-year windows), and the y-axis shows MAE. Lines correspond to MAE values for models trained on entire and specific regions, as indicated in the legend.

Changes in *R*^2^ and MAE values obtained using a sliding 8-year window are presented in Figure 5 and Figure 6, respectively. A similar trend was observed across both metrics. The model trained on the mandible consistently demonstrated the highest overall predictive accuracy within younger ages, but this performance declined around the onset of adulthood and remained lower thereafter. Models trained on the maxilla showed the second-highest predictive accuracy, corresponding to the age ranges of pubertal growth, similar to the mandibular model.

Regarding age-related changes, the model trained on the cervical vertebrae exhibited higher predictive accuracy both before and after the puberty growth peak compared to other age ranges, resulting in a characteristic bimodal pattern in the performance curve.

The model trained on the frontal region demonstrated the highest predictive accuracy before puberty, whereas the cranial base model performed better after the completion of pubertal growth. The model trained on the entire cephalogram showed a trend that approximated the overall average of the region-specific models.

The highest *R*^2^ values for each anatomical region were as follows: 0.785 for the mandible in the 8- to 15-year-old range, 0.736 for the maxilla in the 8- to 15-year-old range, 0.647 for the cervical vertebrae in the 7- to 14-year-old range, 0.467 for the frontal region in the 7- to 14-year-old range, and 0.239 for the cranial base in the 16- to 23-year-old range (Figure 5).

The lowest MAE was observed in the mandible within the 10- to 17-year-old range, at 0.87 years. The age ranges showing the highest predictive accuracy for each region were as follows: 8–15 years for the maxilla (MAE = 0.95 years); 7–14 years for the cervical vertebrae (MAE = 1.13 years); 5–12 years for the frontal region (MAE = 1.46 years); and 17–24 years for the cranial base (MAE = 1.63 years) (Figure 6).

### 3.4. ResNet50 vs. ConvNeXt-T

Both architectures showed similar performance trends across anatomical regions. The model trained on the entire cephalometric image achieved the highest predictive accuracy, while among the region-specific models, the mandible and cervical vertebrae models demonstrated relatively superior performance. However, when comparing the two architectures, ResNet50 consistently outperformed ConvNeXt-T across models trained on the entire image and specific regions. In the <20-year-old group, all local models in the ResNet50 architecture yielded positive R^2^ values, whereas those in the ConvNeXt-T architecture were negative for all local models except the cervical vertebrae model (Table 4). Bootstrap-derived 95% confidence intervals for the differences in MAE between ResNet50 and ConvNeXt-T in the full-image model are presented in Table 5.

### 3.5. Attention Maps of the Mandible and Cranial Base Models

**Figure 7:**
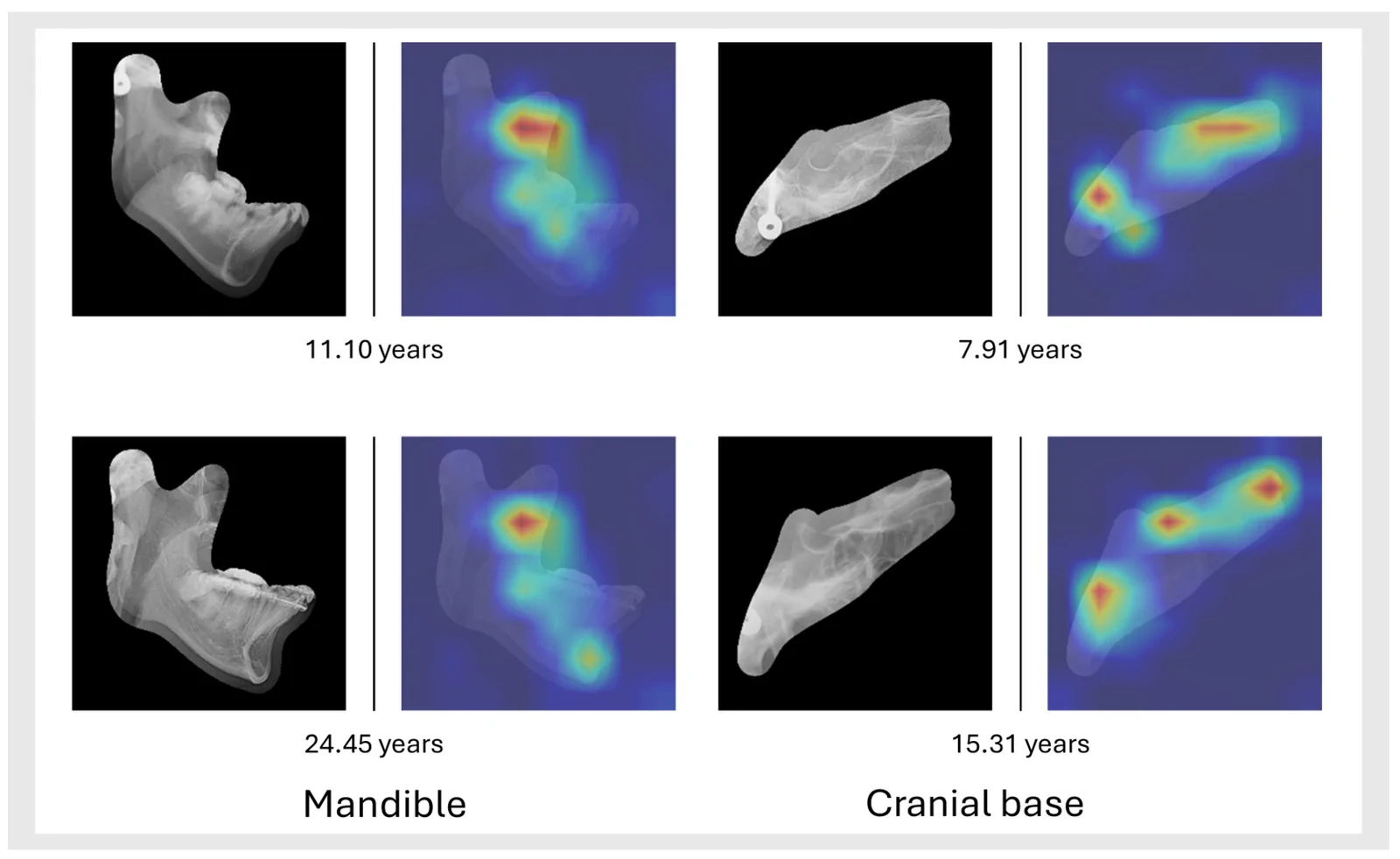
Attention maps of the mandible and cranial base models. Attention maps generated using Grad-CAM++. Heatmaps are overlaid on the input images. Warm colors (red/yellow) indicate regions to which the model pays greater attention, whereas cool colors (blue) indicate regions with low attention. Chronological age (in years) is shown below each image.

Attention maps generated using Grad-CAM++ showed that the mandibular model (the highest-performing region) consistently highlighted developing tooth germs, the mental protuberance, and the anterior border of the mandibular ramus. In contrast, the cranial base model (the lowest-performing region) primarily highlighted areas along the superior orbital wall and around the spheno-occipital synchondrosis (Figure 7).

## 4. Discussion

Overall, the present study demonstrated that the CNN model was able to predict chronological age from cephalometric images with relatively high accuracy during the growth period, although predictive performance declined after completion of growth. Model performance was evaluated using three complementary metrics: MAE; *r*; and *R*^2^. Each of these metrics captures a distinct aspect of predictive performance and has inherent strengths and limitations. A comprehensive evaluation of model validity thus requires the integrated interpretation of all three indicators, rather than reliance on a single metric.

MAE represents the average magnitude of prediction error and provides an intuitive measure of how far estimated age deviates from actual chronological age in a clinically interpretable manner. In the present study, MAE was 1.16 years in the <20-year-old group and 2.73 years in the ≥20-year-old group, indicating substantially lower prediction error in the younger group. Mellion et al. [2], using longitudinal data from 100 subjects, reported that the estimation error for the onset and peak of pubertal growth based on HWM and CVM was approximately 1 year. Cephalograms obtained during the growth period inherently reflect interindividual variability in skeletal maturity and craniofacial morphology. Predicting chronological age from cephalograms while capturing interindividual variability may therefore be more challenging than estimating the timing of pubertal growth. Nevertheless, the magnitude of prediction error observed in the present study remained within a comparable temporal scale. Although the ability to conduct direct comparisons is limited due to differences in study populations, analytical approaches, and evaluation metrics, the level of accuracy may further improve when the model is applied to the prediction of skeletal maturity rather than chronological age.

However, reliance on a single metric, such as MAE, is insufficient for accurately evaluating the prediction of craniofacial growth. Even when MAE is low, *R*^2^ or *r* may remain low, indicating poor agreement in overall trends. MAE reflects only the magnitude of prediction error and so cannot be used to evaluate whether predicted values adequately capture age-related trends.

Pearson’s correlation coefficient (*r*) was therefore used to evaluate the extent to which predicted values reflect the increasing trend in chronological age. Values for *r* were 0.952 in the <20-year-old group and 0.585 in the ≥20-year-old group, indicating a substantially stronger correlation between predicted and actual ages in younger individuals. Because *r* reflects the degree to which chronological and predicted ages vary in the same direction, the higher correlation observed in the <20-year-old group suggests that the model more effectively captures growth-related morphological changes during this period.

Further, the coefficient of determination (*R*^2^) represents the proportion of variance in chronological age explained by each model and was used to evaluate predictive consistency and explanatory power. While MAE reflects the magnitude of prediction error, *R*^2^ evaluates how well the model accounts for variability in actual age. In the present study, *R*^2^ values were positive for all models in the <20-year-old group, indicating that models successfully learned age-related features from cephalograms, although performance varied across regions. In contrast, *R*^2^ values were negative in the ≥20-year-old group, reflecting substantially reduced predictive performance. This finding suggests that extracting age-related features becomes more challenging once morphological changes diminish after the completion of growth.

Because both MAE and *R*^2^ are influenced by the variance structure of the evaluated age ranges, sliding-window analyses were additionally performed using 8-year age intervals encompassing the pubertal growth period. Although the selection of the 8-year window was partly empirical and represents a limitation of the present study, this approach enabled us to examine changes in model performance across different age ranges. The improved performance observed in age ranges corresponding to the pubertal growth phase suggests that the model may capture features associated with peak growth-related morphological changes (Figure 5 and Figure 6).

Among the region-specific models in the <20-year-old group, the relative ranking of model performance varied depending on the evaluation metric used. Based on the values of *R*^2^ and *r*, models were ranked in the following order of accuracy: entire image; mandible; cervical vertebrae; frontal region; maxilla; and cranial base. In contrast, rankings evaluated using MAE were the entire image; mandible; maxilla; cervical vertebrae; frontal region; and cranial base. The maxillary model thus showed a relatively higher ranking (Table 3). This discrepancy can be attributed to the inherent characteristics of the evaluation metrics. MAE provides an intuitive measure of the magnitude of prediction error and is relatively robust to outliers. However, the value is less sensitive to the dispersion of errors and the stability of predictions. In contrast, values of *R*^2^ and *r* more strongly reflect the extent to which the variability in predicted values corresponds to variability in observed values. These findings thus suggest that maxillary growth exhibits substantial interindividual variability in growth timing, which may lead to a higher incidence of outliers and reduced stability of model performance.

The frontal region exhibited comparable predictive accuracy to that of the mandible and cervical vertebrae during the prepubertal stage (Figure 5 and Figure 6). Ruf and Pancherz [13]. reported that morphological changes in the frontal sinus during the growth period correlated significantly with both height growth curves and HWM. Our results are in agreement with previous reports and support the potential role of the frontal region as a novel anatomical indicator for growth assessment.

The mandibular model demonstrated the highest overall accuracy among all region-specific models, maintaining high predictive performance from the prepubertal through post-pubertal stages. This superior performance may be attributable not only to the inclusion of dental-age-related information but also to the fact that the mandible undergoes diverse and dynamic morphological changes throughout the growth period, particularly in comparison with the maxilla.

The cervical vertebrae model demonstrated the second-highest predictive accuracy after the mandible, supporting its established clinical relevance as an indicator of skeletal growth, in agreement with previous studies [4,5,9,10,11]. Notably, the cervical vertebrae model exhibited the highest predictive accuracy after the pubertal growth spurt, suggesting potential utility as a marker for determining the cessation of growth.

Attention map analysis further revealed that in the mandible region, the model focused not only on the developing tooth germs but also prominently on the mental protuberance and the anterior border of the mandibular ramus. These findings suggest that the model primarily captured features associated with bone remodeling for age prediction. Notably, because the mental protuberance continues to undergo morphological changes even after the pubertal growth spurt, the emphasis of the model on this region may partially account for the superior predictive accuracy of the mandibular model compared with other anatomical regions.

In contrast, in the cranial base, which showed the lowest predictive accuracy, the model primarily attended to the superior wall of the orbit and the region surrounding the spheno-occipital synchondrosis. Because growth of the cranial base, particularly the region from the foramen cecum to the pituitary fossa, is known to reach adult dimensions before puberty, the anterior cranial margin remains relatively stable even during the pubertal growth period [21,22,23]. One possibility is that the model utilized this structural stability to distinguish between pre- and post-pubertal stages. However, as indicated by the growth curves described by Scammon [24], the cranial base, which is predominantly influenced by neural growth, reaches skeletal maturation earlier than other craniofacial structures. The age range of the dataset used in this study (5–30 years) may thus have provided insufficient developmental variation for the model to effectively learn informative age-related features, resulting in reduced predictive accuracy even in the prepubertal age groups. In addition, saliency-based visualization methods provide only an approximate representation of the regions associated with model predictions, and the highlighted regions may vary depending on the visualization method and model architecture used. Attention patterns in the present study also showed considerable variability across images. Therefore, further methodological refinement is required before attention maps can be utilized as a standardized evaluation tool.

The primary objective of this study was not to maximize predictive performance for clinical application but rather to investigate whether a CNN could learn growth-related features from cephalograms. Therefore, this study was designed primarily as a proof-of-concept investigation. Chronological age was adopted as the reference label for model training because it provides a continuous variable, allowing a more sensitive evaluation of age-related morphological changes than conventional skeletal maturity indicators based on discrete stages. If chronological age is regarded as the result of random variations added to fundamental skeletal maturation, the predictions generated by the present model may more closely approximate the underlying skeletal maturity [25]. As long as individual variability as an inherent uncertainty is included in the target data (chronological age), chronological age is inherently difficult to predict from cephalograms with perfect accuracy. However, through training processes using a large volume of age-labeled data, AI models may statistically eliminate such random variations derived from individual differences, suggesting that the models may preferentially capture the consistent, growth-related component of the morphological signal. This concept is analogous to the “Noise2Noise” learning method in AI [26]. In this approach, a model can learn to reconstruct the original noise-free signal even without access to clean ground-truth images. This is achieved by training on multiple images of the same underlying data, each corrupted by different types of noise. Similarly, in the present study, by learning chronological age values that include random variations added to fundamental skeletal maturation, the model may have captured consistent morphological patterns that are associated with skeletal maturation rather than chronological age alone. However, the interpretation that these findings may be applicable to skeletal maturation assessment remains hypothetical at present and requires further validation in future studies.

ResNet50 and ConvNeXt-T showed similar performance trends across anatomical regions. The model trained on the entire cephalometric image achieved the highest predictive accuracy, while among the region-specific models, the mandible and cervical vertebrae models demonstrated relatively superior performance. These findings support the above discussion that the mandible and cervical vertebrae may contain more informative growth-related features than other craniofacial regions. The ResNet50 architecture used in this study is a CNN capable of effectively processing high-dimensional and complex information, such as medical images. ResNet50 incorporates residual connections, also known as skip connections, into the deep network structure to mitigate the vanishing gradient problem and is particularly effective at capturing grayscale variations that reflect bone quality and the spatial relationships among anatomical structures. In contrast, ConvNeXt is a more recent CNN architecture that modernizes the original ResNet design by incorporating elements inspired by Vision Transformers, such as 7 × 7 depthwise convolutions, layer normalization, and GELU activations, to more efficiently capture broader contextual information. Previous studies have reported that ConvNeXt demonstrates excellent performance in large-scale models [27]. As a result, ConvNeXt-T was considered suitable to meet the objectives of this study, in which both fine-grained changes in bone morphology and global craniofacial structure contribute to age estimation. However, ConvNeXt is relatively sensitive to hyperparameter settings and training strategies. The primary objective of this study was not to maximize predictive performance for clinical application, but rather to investigate whether a CNN could learn growth-related features from cephalometric images. Therefore, early stopping using a validation dataset and large-scale hyperparameter optimization aimed at maximizing prediction accuracy were not performed. As a result, it is possible that the hyperparameter settings used in the present study were not fully optimal for ConvNeXt-T. In addition, compared with natural images, medical imaging datasets are often limited in size. Consequently, under the conditions of the present study, ConvNeXt-T may not have been able to fully realize its performance potential.

The present study has several limitations that must be considered when attempting to interpret the results. One important limitation was that a cross-sectional design was employed because longitudinal data were not included in order to secure a sufficient dataset. As demonstrated in previous growth studies [28,29,30,31], longitudinal study designs are more appropriate for rigorously analyzing the interindividual differences in growth direction and magnitude that characterize facial patterns. However, a cross-sectional investigation by Hans reported that histological specimens from the posterior and anterior borders of the mandibular ramus showed characteristic and consistent patterns of bone resorption and apposition [32]. If such “site-specific remodeling patterns” are associated with individual facial morphology (facial pattern [29]), the models trained in the present study may have learned facial-pattern–related features to some extent even from a cross-sectional dataset, potentially influencing prediction accuracy.

Another limitation of this study was the uneven distribution of cases across age groups. Although the overall cohort size was sufficient, some age groups, particularly in the cranial base model, offered only limited training and testing data, which may have affected prediction accuracy. In addition, this study was conducted as a retrospective analysis of patients with malocclusion. The growth patterns observed thus may not be fully representative of individuals with normal occlusion and typical craniofacial development.

Because the present study was conducted primarily as a proof-of-concept investigation and did not include a validation dataset or external validation, immediate clinical application of the proposed model remains difficult. However, with the incorporation of more diverse age groups and longitudinal datasets, the generalizability and clinical reliability of the model could be further validated. Further, the model developed in the present study provides a foundation for application to other tasks related to craniofacial growth analysis. One potential approach is transfer learning, a machine learning method that applies knowledge obtained from a previously trained model to another related task. Using the present model as a pretrained network for transfer learning, and by reintroducing interindividual variability and other sources of noise, future cephalograms similar to a visual treatment objective may potentially be generated [29].

## 5. Conclusions

This study demonstrated that a CNN-based model could predict chronological age from cephalograms with high accuracy, particularly in patients <20 years old. The model may have extracted age-associated morphological features related to craniofacial growth; this interpretation is supported by the finding that prediction was most accurate in regions clinically used to assess growth (notably the mandible and cervical vertebrae) and that the model attended to sites of active bony remodeling. These findings suggest the potential utility of AI-based cephalometric analysis for skeletal maturation research and the investigation of age-related craniofacial morphological patterns. As a proof of concept, this approach may provide a foundation for future studies that aim to predict craniofacial growth through transfer learning. However, further validation and improvement of the model are required before clinical application in orthodontic practice.

## Figures and Tables

**Table 1:** Sample sizes and age distributions of the <20-year and ≥20-year subgroups in the test dataset. The table summarizes the numbers of radiographs and patients, together with the mean age and standard deviation, in each subgroup. Patients could be included in both age subgroups if multiple cephalograms were available at different ages.

Group	Radiographs (n)	Patients (n)	Mean Age ± SD
<20 years	58	37	12.77 ± 4.28
≥20 years	44	30	25.35 ± 3.10

**Table 2:** Sex Distribution of the Sliding 8-Year Age Groups. The figure summarizes the demographic composition of the sliding 8-year age windows used to explore performance trends across different age ranges. Each subgroup was generated by shifting the age range by one year, and the numbers of male and female subjects included in each subgroup are presented.

Age Range (Years)	Male	Female	Total
5–12 years	11	9	20
6–13 years	12	11	23
7–14 years	11	11	22
8–15 years	10	14	24
9–16 years	9	15	24
10–17 years	7	14	21
11–18 years	7	14	21
12–19 years	5	16	21
13–20 years	6	16	22
14–21 years	6	16	22
15–22 years	6	15	21
16–23 years	6	15	21
17–24 years	4	16	20
18–25 years	5	13	18
19–26 years	5	14	19
20–27 years	5	15	20
21–28 years	6	16	22
22–29 years	7	16	23

**Table 3:** Performance of the ResNet50 model. The table shows mean absolute error (MAE), Pearson’s correlation coefficient (*r*), and coefficient of determination (*R*^2^) with 95% confidence intervals (CIs) for subjects <20 years old and ≥20 years old. * *p* < 0.05, ** *p* < 0.001 for the correlation coefficient *r* between chronological age and predicted age.

		MAE		*r*		*R* ^2^	
		<20 Years (95% CI)	≥20 Years (95% CI)	<20 Years (95% CI)	≥20 Years (95% CI)	<20 Years (95% CI)	≥20 Years (95% CI)
Entire image		1.17 (0.95–1.41)	2.73 (2.16–3.34)	0.952 **	0.585 **	0.884	−0.221
				(0.930–0.971)	(0.347–0.756)	(0.829–0.921)	(−0.925–0.211)
Frontal		1.79 (1.48–2.10)	4.57 (3.84–5.32)	0.913 **	0.382 *	0.741	−1.925
region				(0.878–0.943)	(0.104–0.640)	(0.596–0.828)	(−3.382– −1.105)
Cranial bone		2.77 (2.29–3.31)	3.81 (2.94–4.65)	0.838 **	0.237	0.362	−1.360
				(0.759–0.895)	(−0.030–0.501)	(0.037–0.569)	(−2.640– −0.606)
Maxilla		1.52 (1.09–2.03)	3.22 (2.53–3.94)	0.877 **	0.445 **	0.709	−0.690
				(0.794–0.943)	(0.188–0.647)	(0.497–0.853)	(−1.695– −0.109)
Mandible		1.17 (0.96–1.39)	3.15 (2.55–3.82)	0.951 **	0.492 **	0.883	−0.515
				(0.933–0.968)	(0.244–0.701)	(0.836–0.918)	(−1.342– −0.058)
Cervical vertebrae		1.59 (1.29–1.90)	2.78 (2.21–3.40)	0.944 **	0.605 **	0.774	−0.227
				(0.923–0.966)	(0.372–0.770)	(0.659–0.848)	(−0.959–0.193)

**Table 4:** Comparison of ResNet50 and ConvNeXt-T models. The table shows mean absolute error (MAE), Pearson’s correlation coefficient (*r*), and coefficient of determination (*R*^2^) for subjects <20 years old and ≥20 years old. * *p* < 0.05, ** *p* < 0.001 for the correlation coefficient *r* between chronological age and predicted age.

	MAE				*r*				*R* ^2^			
	<20 Years		≥20 Years		<20 Years		≥20 Years		<20 Years		≥20 Years	
	ResNet	ConvNeXt	ResNet	ConvNeXt	ResNet	ConvNeXt	ResNet	ConvNeXt	ResNet	ConvNeXt	ResNet	ConvNeXt
Entire	1.17	1.59	2.73	3.80	0.952 **	0.896 **	0.585 **	0.30 **	0.884	0.781	−0.221	−1.552
image												
Frontal	1.79	6.54	4.57	4.16	0.913 **	0.093	0.382 *	0.210	0.741	−2.404	−1.925	−1.72
region												
Cranial bone	2.77	4.09	3.81	5.25	0.838 **	0.611 **	0.237	0.191	0.362	−0.469	−1.360	−2.822
Maxilla	1.52	5.13	3.22	4.11	0.877 **	0.496 **	0.445 **	0.124	0.709	−1.143	−0.690	−1.985
Mandible	1.17	4.64	3.15	3.68	0.951 **	0.675 **	0.492 **	0.032	0.883	−0.779	−0.515	−1.067
Cervical vertebrae	1.59	2.21	2.78	4.80	0.944 **	0.867 **	0.605 **	0.218	0.774	0.573	−0.227	−2.601

**Table 5:** Statistical comparison between the ResNet50 and ConvNeXt-T models. The table presents the predictive performance of the ResNet50 and ConvNeXt-T models for chronological age estimation from cephalometric images. Mean absolute error (MAE) values are presented with 95% confidence intervals. Differences between the two architectures (ConvNeXt-T–ResNet50) are also shown.

	ResNet50 MAE (95% CI)	ConvNeXt-T MAE (95% CI)	ResNet50–ConvNeXt-T (95% CI)
<20 years	1.17 (0.95–1.41)	1.59 (1.29–1.94)	−0.426 (−0.737– −0.107)
≥20 years	2.73 (2.16–3.34)	3.80 (2.98–4.73)	−1.067 (−1.627– −0.517)

## Data Availability

The data presented in this study are not publicly available due to ethical and privacy restrictions.

## Abbreviations

The following abbreviations are used in this manuscript:

HWM	hand–wrist maturation
CVM	cervical vertebral maturation
CNN	convolutional neural network
AI	artificial intelligence
